# Evolution of DDB1-binding WD40 (DWD) in the viridiplantae

**DOI:** 10.1371/journal.pone.0190282

**Published:** 2018-01-02

**Authors:** Rahul Tevatia, George A. Oyler

**Affiliations:** 1 Department of Chemical and Biomolecular Engineering, Johns Hopkins University, Baltimore, Maryland, United States of America; 2 Synaptic Research LLC, Baltimore, Maryland, United States of America; National Taiwan University, TAIWAN

## Abstract

Damaged DNA Binding 1 (DDB1)—binding WD40 (DWD) proteins are highly conserved and involved in a plethora of developmental and physiological processes such as flowering time control, photomorphogenesis, and abiotic stress responses. The phylogeny of this family of proteins in plants and algae of viridiplante is a critical area to understand the emergence of this family in such important and diverse functions. We aimed to investigate the putative homologs of DWD in the viridiplante and establish a deeper DWD evolutionary grasp. The advancement in publicly available genomic data allowed us to perform an extensive genome-wide DWD retrieval. Using annotated *Arabidopsis thaliana* DWDs as the reference, we generated and characterized a comprehensive DWD database for the studied photoautotrophs. Further, a generic DWD classification system (Type A to K), based on (i) position of DWD motifs, (ii) number of DWD motifs, and (iii) presence/absence of other domains, was adopted. About 72–80% DWDs have one DWD motif, whereas 17–24% DWDs have two and 0.5–4.7% DWDs have three DWD motifs. Neighbor-joining phylogenetic construction of *A*. *thaliana* DWDs facilitated us to tune these substrate receptors into 15 groups. Though the DWD count increases from microalgae to higher land plants, the ratio of DWD to WD40 remained constant throughout the viridiplante. The DWD expansion appeared to be the consequence of consistent DWD genetic flow accompanied by several gene duplication events. The network, phylogenetic, and statistical analysis delineated DWD evolutionary relevance in the viridiplante.

## Introduction

The central hypothesis for viridiplantae ancestry involves diversification of the early flagellates into two clades: i) chlorophyta (prasinophytes granting the core chlorophytes), and ii) streptophyta (charophytes evolving to the early land plants) [1,2]. The ubiquitin cascade provided one of the immediate surveillance for the photoautotrophic survival. The ubiquitination process regulates several functions in plants, such as circadian clock control, abiotic stress responses, environmental signal transduction, histone H2A monoubiquitination, mitotic cell cycle control, down-regulation of apoptosis, and virus regulation [3,4].

In proteasomal three-enzyme ubiquitination system, E1 enzyme initiates the process via ATP-dependent ubiquitin activation, followed by the transfer of activated ubiquitin to E2 enzyme. The E3 ubiquitin ligase binds to the activated E2 at the C-terminal and the specific substrate at the N-terminal to perform final catalytic ubiquitination event [5,6]. The CULLIN-RING LIGASES (CRLs) comprise the largest known class of E3 ubiquitin ligases family. A typical CRL (multi-protein complex) has two modules attached to the cullin protein: a) RING (Really Interesting New Gene) finger protein (Rbx1/ROC1/Hrt1) attached to the C-terminal and b) the substrate receptor at the N-terminal [7–9]. Cullin-RING interacts with various linker proteins to recruit a substrate receptor. For instance, CUL1 and 7 binds to SUPPRESSOR OF KINETOCHORE PROTEIN (SKP1)-F-BOX, CUL2 and 5 binds to ELONGIN BC-BC-BOX- SUPPRESSOR OF CYTOKINE SIGNALING (SOCS), CUL3 binds to BRIC-A-BRAC, TRAMTRACK and BROAD (BTB)-domain protein, and CUL4A binds to DAMAGED DNA BINDING PROTEIN 1 (DDB1) [10].

**Fig 1:**
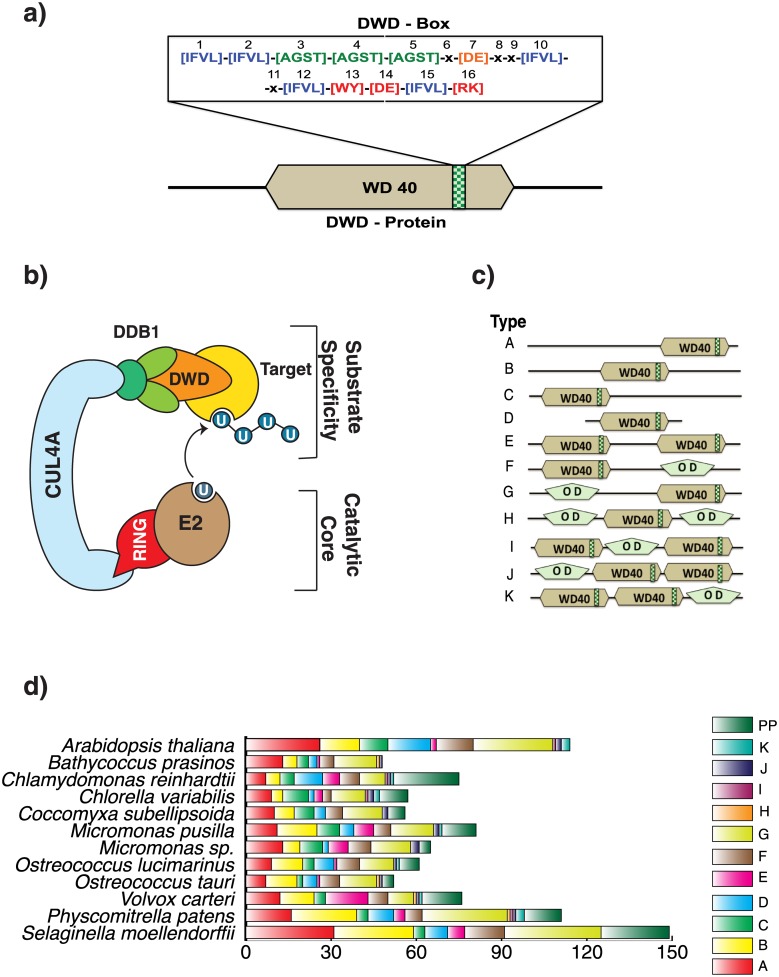
DWD in the viridiplante. a) A typical DWD motif with an arrangement of highly conserved 16 amino acids. b) A schematic representation of CUL4A-RING-DDB1 E3 ligase. The unit includes Cul4 connected to RING at the C-terminal (which links the activated E2 enzyme) and DDB1 at the N-terminal (which binds DWD). c) Generic classification system, Type A-K, to differentiate DWDs based on (i) position of DWD motif and (ii) number of DWD motif and (iii) presence or absence of other domains. d) The statistical variation in DWD types. (PP—partial proteins).

The insight in CUl4A-DDB1-RING complex architecture resolved the mechanism of DDB1 binding to the DWD protein [11], which in turn can be exploited to identify a DWD. A DWD motif (also known as WDxR or DxR, Fig 1a) is unique signature in DWD proteins that provides a binding site for DDB1 [12]. DDB1 forms a multidomain structure consisting of three 7-bladed beta-propellers (referred to as BPA to BPC for Beta-propeller A to C) and functions as an adaptor protein bridging CUL4A and a substrate receptor. The Beta Propeller A-C (BPA-BPC) folds tightly and presents a substrate specific binding pocket at the N-terminal of CUL4A (Fig 1b) [13]. DDB1 is structurally flexible with three different structural states [12–15] and presents an optimal orientation for the substrate to accept ubiquitin from the E2 ubiquitin-conjugating enzyme to complete the ubiquitination.

WD40 proteins have several copies of 40–60 residues repeats ending with Trp-Asp (WD) at the C-terminal. Each WD 40 repeat embraces a four-stranded antiparallel beta sheets [16–19]. Experimental studies and bioinformatics analysis concluded that DWDs are the subset of WD40 proteins (about one-third of WD40 proteins are DWDs) [20,21]. Therefore, WD40 are the targets to mine DWD. Multiple sequence alignment and DWD structural analysis revealed a conserved 16 amino acids long DWD motif (WDxR or DxR) [20]. The motif includes hydrophobic residues (Ile/Phe/Val/Leu) at 1, 2, 10, 12, and 15^th^ positions, and small amino acids (Ala/Gly/Ser/Thr) at 3, 4, and 5^th^ positions [20]. Most important and highly conserved residues include Asp or Glu—7, Trp or Tyr—13, Asp or Glu—14, and Arg or Lys—16. The Arg-16 is bottom faced to interact with DDB1 [21]. The mutational studies on Arg or Lys at 16^th^ position of DWD motif resulted in a weak DDB1-DWD binding [12,22], indicating the Arg/Lys significance in the motif.

Till date, a total of 85 in *Arabidopsis* [23], 78 in rice (*Oryza sativa*) [23] and 161 in soybean (*Glycine max*) [24] putative DWD proteins have been identified using *in silico* methods and characterized in plants, and some of the important putative DWDs were confirmed using protein-protein interaction assays. However, in Virdiplantae, a broad spectrum of DWD proteins to understand the emergence of this family of proteins is still unclear. Besides how evolutionary convergence or divergence of DWD is linked to various cellular and developmental processes has not been explored. Herein, we performed a genome-wide retrieval of DWD proteins in nine microalgae, one fern, one moss, and one angiosperm. We characterized DWDs keeping *Arabidopsis thaliana* annotated database as the reference. Finally, we extended the green ancestry based on these highly conserved DWD proteins and studied the relevance of conservation of DWDs in the viridiplante.

## Materials and methods

### DWD homologs mining

This study includes genome sequenced and annotated microalgae—*Bathycoccus prasinos* (NCBI TaxID—41875), *Chlamydomonas reinhardtii* (NCBI TaxID—3055), *Chlorella variabilis* (NCBI TaxID—554065), *Coccomyxa subellipsoida* (NCBI TaxId—248742), *Micromonas pusilla* (NCBI TaxID—38833), *Micromonas sp* (NCBI TaxID—296587), *Ostreococcus lucimarinus* (NCBI TaxID—242159), *Ostreococcus tauri* (NCBI TaxID—70448), *Volvox carteri* (NCBI TaxID—3067), angiosperm -*Arabidopsis thaliana* (NCBI TaxID—3702), bryophyte—*Physcomitrella patens* (NCBI TaxID—3218), and lycophyte—*Selaginella moellendorffii* (NCBI TaxID—88036). The respective annotated genomic data was acquired from the public domain National Centre for Biotechnology Information (http://www.ncbi.nlm.nih.gov).

WD40 proteins were retrieved using the Domain Enhanced Lookup Time Accelerated Blast (DELTA-BLAST, NCBI) [25] at an e-value cutoff of E-10 and confirmed with WD40 repeat protein Structure Predictor (WDSP) [26]. Each WD40 protein was then scrutinized for DWD motif [IFVL]-[IFVL]-[AGST]-[AGST]-[AGST]-x-[DE]-x-x-[IFVL]-x-[IFVL]-[WY]-[DE]-[IFVL]-[RK] using an in-house PERL script.

### Characterization of DWD homologs

The DWD database for *A*. *thaliana* was kept as a reference to characterize other DWD proteins. DWDs (retrieved in other viridiplante) were locally BLASTp against *A*. *thaliana* DWD database. Based on percent similarities of Blast results, a heat map was constructed to enrich DWDs.

### DWD multiple sequence alignment

Since the protein domains are highly specific with conserved biological functions compared to the entire protein [27], the multiple sequence alignment for DWD motifs was executed using ClustalW [28]. The gap opening penalty was reserved to 10 with an extension penalty of 0.2. The Gonnet protein weight matrix, with a delay divergent cutoff of 30%, was implemented.

### Phylogenetic construction

The phylogenetic tree was constructed using MEGA 7.0.20 [29]. The neighbor-joining method [30] was executed with 1,000 replicates using bootstrap test [31]. The evolutionary distances (the units of the number of amino acid differences per site) were computed using the p-distance method. Two phylogenetic trees were created with: (i) all DWDs characterized in the viridiplante, and (ii) DWDs homologs common to the viridiplante lineage.

### Network visualization

The functional association of *A*. *thali*ana DWDs was performed using STRING ver 10.0 [32]. The nodes represent DWDs, and edges denote the interaction. The prediction score was kept at high confidence (0.7) to restrict the association map only to the significant interactions. The edge thickness represents the strength of an interaction, where more thickness implies a strong interaction. The networking is based on deterministic spring model while the position of nodes indicates the minimum energy achieved for the system.

## Results

### Identification and characterization of DWD proteins

To identify DWD protein homologs in the viridiplante lineage, first we searched WD40 proteins in the database of nine chlorophyte microalgae (*Bathycoccus prasinos*, *Chlamydomonas reinhardtii*, *Chlorella variabilis*, *Coccomyxa subellipsoida*, *Micromonas pusilla*, *Micromonas sp*., *Ostreococcus lucimarinus*, *Ostreococcus tauri*, *Volvox carteri*), one angiosperm (*Arabidopsis thaliana*), one bryophyte (*Physcomitrella patens*), and one lycophyte (*Selaginella moellendorffii*). Then, we scrutinized each WD40 for DWD protein using an in-house PERL programming script.

Based on the position of WD40 repeats and the presence/absence of other domains, we grouped DWD proteins in eleven different types—A to K (Fig 1c). Detailed list and information on all identified other-domains are compiled in S1 Table. The most common types are Type G (~12–31% of all DWDs), followed by Type A (~ 10–28%), Type B (~12–21%), and Type F (~ 5–14%), whereas Type H to K have negligible DWD counts (< 2%). This statistical analysis is consistent throughout the studied photoautotrophs (Fig 1d).

**Fig 2:**
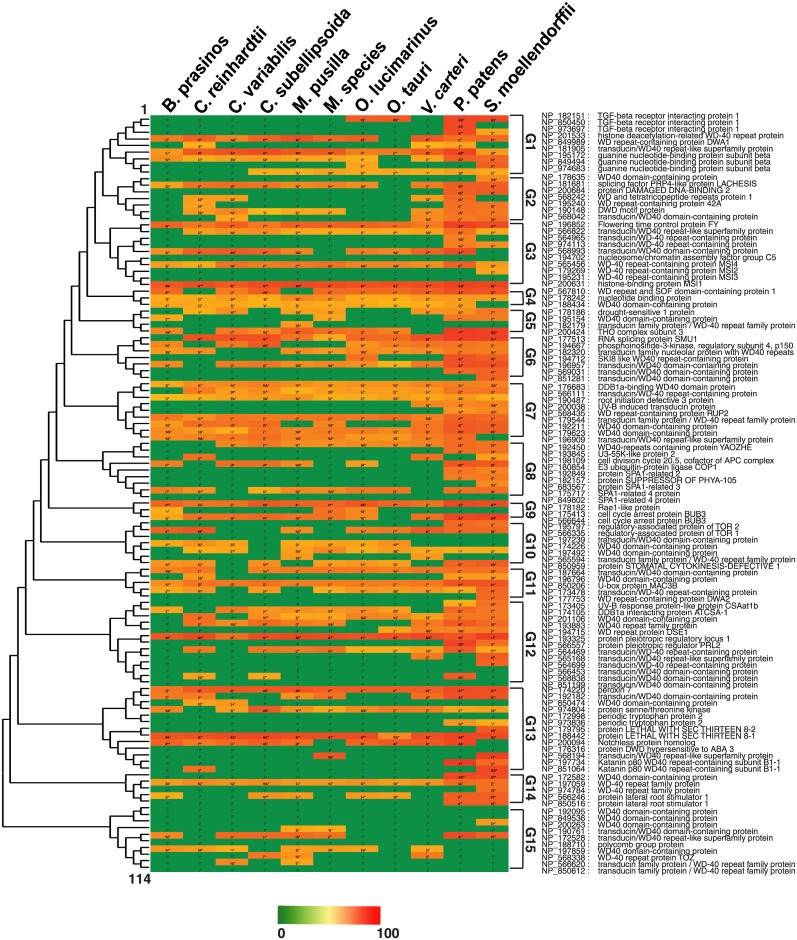
Identification and characterization of DWD. The predicted DWD in each family was characterized using *A*. *thaliana* annotated DWD database. The heat map illustrates the percent similarity of query DWD obtained after BLASTp with *A*. *thaliana* DWD database. The phylogenetic tree was constructed using neighbor joining method and *A*. *thaliana* DWD proteins were distributed into 15 groups.

To characterize DWD proteins, we created *A*. *thaliana* DWD database (S2 Table) and designated it as the reference. The DWD list with respective motif sequences are compiled in S1 File. We performed BLASTp for each identified microalgal/land plant DWD protein with *A*. *thaliana* DWD database (S3 Table). The percent similarity was recorded for the hits (S4 Table), and used to generate a heat chart (Fig 2) arranged as per evolutionary arrangement of *A*. *thaliana* DWD proteins (S1 Fig). *A*. *thaliana* DWD based phylogenetic tree convened DWD proteins into 15 distinct groups. At-least one DWD in each group has its corresponding homolog in the lineage. Further, heat map allowed us to measure the orthology in these 15 groups. G7 has the highest percent of homologs (~ 77.8%) whereas G15 has the least (~20%).

### DWD in various pathways

**Fig 3:**
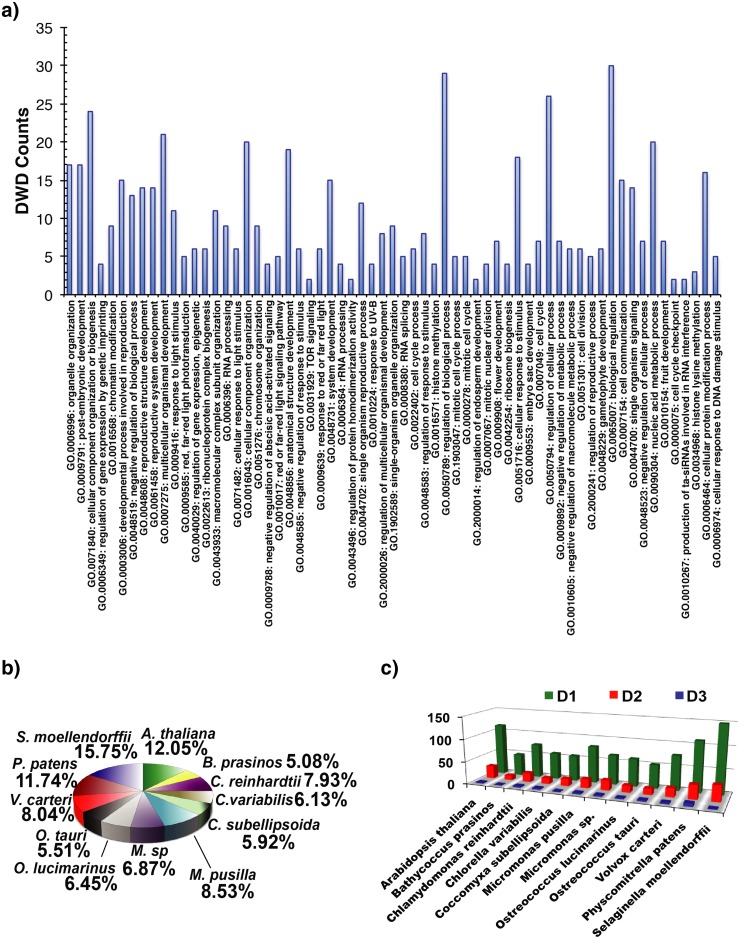
Functional pattern and statistical distribution of DWD. a) The histogram depicting the number of DWD counts in *A*. *thaliana* distributed in various functional pathways. Each pathway has an accession ID with a short description. b) The distribution of DWD in the studied photoautotrophs. c) The statistical variation in the counts of D1, D2 and D3 DWD motifs.

To understand the involvement of DWD in different pathways, we arranged *A*. *thaliana* DWD based on respective functional annotation. DWDs are related to most of the cellular traits, where the DWD distribution in various pathways is extensive (Fig 3a). We observed a higher DWD count for the processes involved in organelle organization, postembryonic development, biogenesis, anatomical structural development, regulation of biological process, cellular response to various stimulus, cell communication, single organism signaling, nucleic acid metabolism process, and cellular protein modification. DWD counts were less, however, for regulation of TOR signaling, protein dimerization activity, endosperm development, cell cycle checkpoint, and RNA interference. Pathways with moderate DWD counts include DNA damage stimulus, photomorphogenesis, carbohydrate metabolism, and photoreception.

### The dynamics of DWD proteins

The DWD counts are species specific (Fig 3b). Irrespective of the variation in WD40 and DWD counts (WD40 counts ranged from 117 to 378, whereas DWD counts ranged from 59 to 185), the DWD pool in WD40 remained uniform during the evolution. Interestingly, DWD to WD40 ratio in microalgae (~57%) is slightly higher than the land plants (~53%) indicating WD40 expanded rigorously compared to DWD with evolution.

To identify the number of DWD motifs required for CUL4A-RING based E3 ubiquitination process, we analyzed the number of DWD motifs in each DWD. DWDs with one motif are 72–80% of total DWD proteins, whereas DWDs with two and three motifs are 17–24% and 0.5–4.7%, respectively (Fig 3c). All species dominate the single domain DWDs and therefore, indicate that even a single copy of DWD motif is adequate for the substrate binding.

### DWD functional network analysis

**Fig 4:**
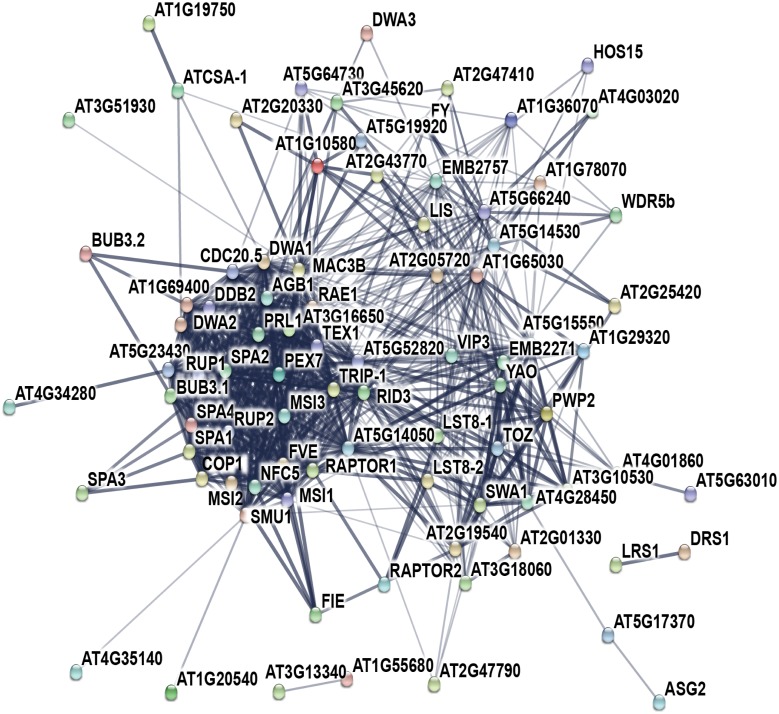
Functional *A*. *thaliana* DWD network. The nodes are DWDs, whereas the edges represent the association. The thickness in edges signifies the strength of confidence score.

To understand the correlation between DWD functional association and evolutionary conservation, we constructed a DWD based protein-protein network for *A*. *thaliana* (Fig 4) and mapped with its corresponding phylogenetic *A*. *thaliana* DWD grouping (Fig 2). The high confidence scores restricted the association to display only the stronger and relevant interactions. The DWD network summarized the significant functional variations among DWDs.

The phylogenetic based DWD grouping and functional based DWD association network reflects a similar pattern. For instance, COP1, SPA1, 2 and 3 are grouped together in G8 with G9 (BUB3 and Rae1) (Fig 2). A similar cluster of SPA and COP1 proteins connected with BUB3 and Rae 1 was observed in the association map (Fig 4). Likewise, G1 (TGF1, DWA1), G2 (DDB2) and G3 (MSI 1,2,3 and 4) are tightly connected in both phylogenetic tree and functionally associated network. MSI and DWA families are prominent in epigenetic controlling of gene expression and abiotic stress tolerance [33,34].

DAMADGED DNA BINDING 2 (DDB2) and *ARABISOPSIS THALIANA* COCKAYNE SYNDROME FACTOR A-1 (ATCSA-1) are specific to DNA repair mechanism but have different mode of actions. Therefore, DDB2 and ATCSA-1 pair is weakly linked in the functional association network [35,36]. Similarly, VERNALIZATION INDEPENDENCE PROTEIN 3 (VIP3) and FY work in a partial antagonistic manner [15] and therefore, this pair is far positioned in the network.

### DWD based viridiplante phylogeny

**Fig 5:**
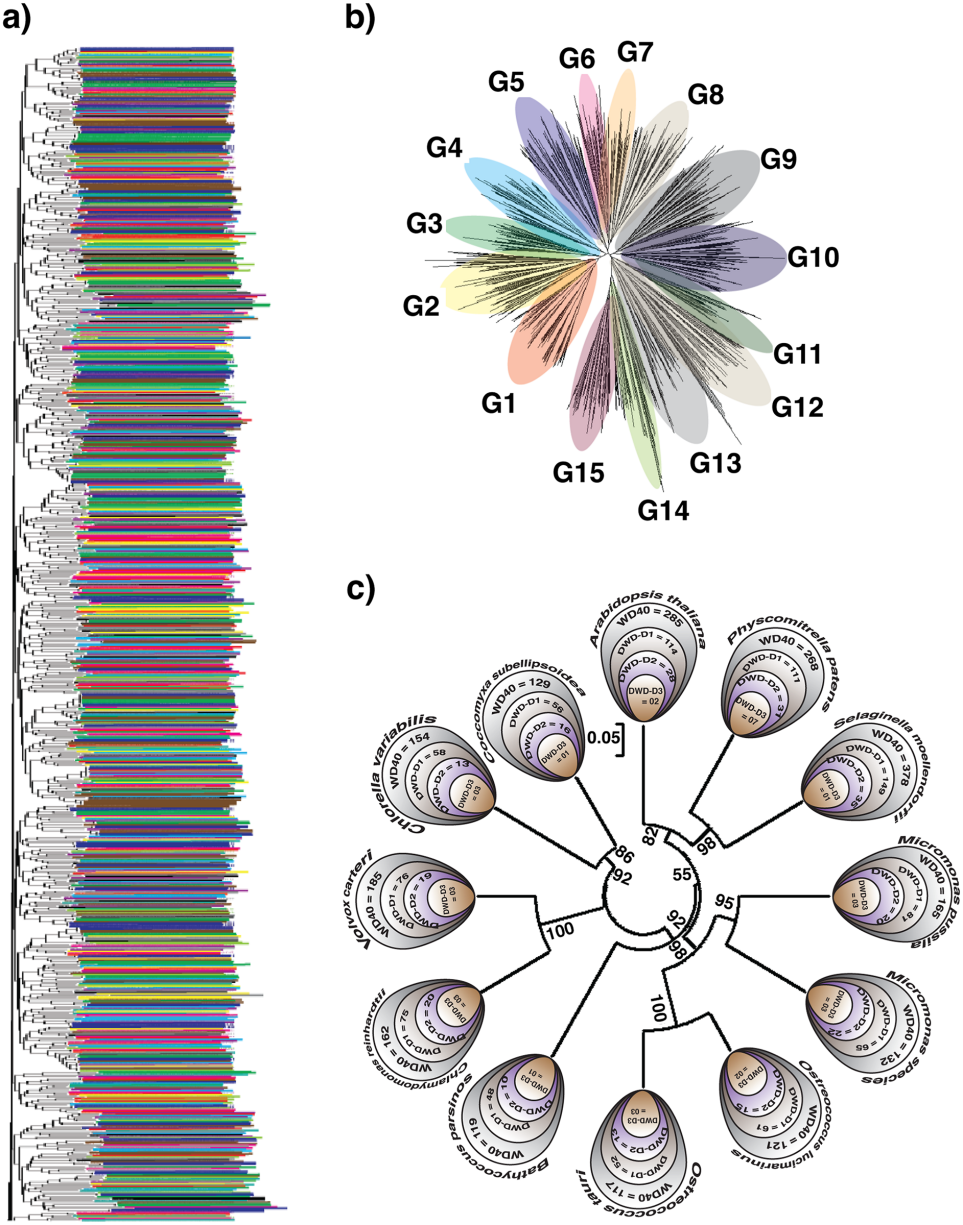
The viridiplante phylogeny. The phylogeny was constructed using neighbor joining method with 1,000 bootstrap replicates. a) Phylogeny of all predicted DWD depicting its random distribution in the lineage. b) The rearrangement of phylogenetic tree build from all DWD and displaying 15 distinct groups. c) Phylogeny reconstruction of viridiplante based on common DWD proteins with statistical analysis at the nodes.

To understand the evolution of DWD proteins, we performed a comprehensive phylogenetic analysis. The distribution of the DWD proteins was random along the phylogenetic tree (the color pattern is non-uniform–Fig 5a). Further, a high conservation of DWD can be seen among the species (Zoom-in of the evolutionary profile is S2 Fig). Further, this tree depicts 15 distinct groups (Fig 5b), very similar to *A*. *thaliana* DWD based phylogenetic groups (Fig 2). Further, we identified 15 mutual DWDs in microalgae and land plants. Using these common DWDs, we constructed a neighbor joining (NJ) phylogenetic tree (Fig 5c). This phylogenetic tree is similar to rRNA or other proteins based phylogenetic constructions [37,38], where land plants (angiosperm, moss and fern) are rooted to microalgae.

## Discussion

We extended the previous knowledge on DWD to identify and characterize them in microalgae and land plants. We mapped all DWDs and reconstructed the viridiplantae lineage evolutionary tree. The current work provides an extensive DWD portfolio allowing exploration of the CUL4-RING E3 ligase-based ubiquitination in viridiplantae.

### Generic DWD classification and expansion of DWD

Jin et al., 2006 [39] and Lee et al., 2008 [23] grouped DWD based on the structure of DWD proteins. Our extensive search for DWD accounts for 88 other domains in various DWDs and cannot be accommodated in previously designed grouping system. Clearly, we needed a different structure to categorize DWD based on a) position of DWD motif, b) number of DWD motifs, and c) presence/absence of other domains. We divided DWD in 11 generic types. Types “A” to “D” have proteins with different positions of DWD motifs. Type "E" has more than one DWD motifs. Types "F" to "K” were created based on other domains that affects the overall structure of DWD proteins.

Next, we searched prokaryotic domains in DWD proteins. *A*. *thaliana*, *C*. *reinhardtii*, and *V*. *carteri* have GrpE homolog (Nucleotide exchange factors for DnaK-type Hsp70s) that function as a co-chaperone in bacteria [40,41]. FliJ (FLAGELLAR BIOSYNTHESIS CHAPERONE)—associated with bacterial flagella and involved in chemotactic responses [42]—was observed in the DWD of *S*. *moellendorffii*. Similarly, GYD (uncharacterized bacterial protein) was part of DWD protein in *B*. *parsinos*.

Microalgae have approximately one-third single domain DWDs (~ 37–40%) [43,44], whereas higher land plants have equal number of single and multi domain DWDs (~ 50–63%) [45,46]. This indicates that DWD gained single domains and lost multi-domains with increment in the evolutionary level. In early microalgae, a higher number of multi-domain DWDs than single domains might be contributed by horizontal gene transfer from prokaryotes (a majority of the bacterial proteins have more than one domains) [47], followed by the DWD gene family expansion. Though the number of DWDs increased randomly during evolution, the ratio of DWDs to WD40s remained constant in all lineages, indicating that the DWD expansion started at early stages of viridiplantae evolution.

### DWD conservation in the viridiplantae lineage

At-least one *Arabidopsis* DWD is evolutionary conserved in each group. For instance, out of nine DWD in G1, four DWD are conserved in microaglae—one TUMOR GROWTH FACTOR β RECEPTOR 1 (TGF-β), one HISTONE DEACYLATION RELATED WD40, and two GUANINE BINDING PROTEINS. Likewise, STOMATAL CYTOKINESIS DEFECTIVE 1 (SCD1) protein—required for cytokinesis and cell expansion in *A*. *thaliana* [48]—is conserved in G10. However, we also observed few DWD strictly restricted to *A*. *thaliana*, like POLYCOMB GENE proteins (G15)—important in reproduction [49], with no respective homolog in microalgae, moss or fern.

The DWD conservation and phylogenetic grouping allowed us to predict the functions of un-annotated homologs (unknown homologs that have not been annotated). FLOWERING TIME CONTROL Y protein (FY), highly conserved DWD and well annotated in Arabidopsis, interact with RNA-binding protein FCA (nuclear RNA binding protein) and control the accumulation of FLOWERING LOCUS C (FLC) [50]. The homologs of *Arabidopsis* FY are un-annotated in other genera of the lineage.

COP1 (CONSTITUTIVE PHOTOMORPHOGENESIS) is a nuclear WD40 that accumulates in dark, fades away in light, and represses the photomorphogenic development [51]. The complex of COP1 and SPA 1 (SUPPRESSOR OF PHYA 1) is a key negative regulator in the light signaling [52]. While COP1 is present in all eukaryotes, SPA proteins are strictly restricted to the plants [53]. We observed evolutionary conservation of COP1 and SPA1 in both microalgae and land plants. Higher plants have two to four paralogs of SPA but all microalgal species have only one SPA homolog.

DWDs involved in the cell cycle regulation were also conserved throughout the lineage. For instance, G9 includes BUB3 (cell cycle arrest protein) and RAE1 (RNA export protein). BUDDING UNIHIBITED BY BENZIMIDAZOLES 3 (BUB3) proteins are WD40 protein involved in mitotic checkpoint at anaphase to yield a kinetochore protein complex capable of delaying anaphase and aid in proper alignment of chromosomes [54]. Ribonucleic Acid Export 1 (RAE-1) have mRNA and ubiquitin binding functions [55].

PLEIOTROPIC REGULATORY LOCUS 1 (PRL1) is a substrate receptor of CUL4-ROC1-DDB1 E3 ligase and degrades *ARABIDOPSIS* KINASE HOMOLG (AKIN) 10 and 11 in *Arabidopsis*. PRL1 encodes for nuclear WD40 and aid in pleiotropic regulation of glucose and hormonal responses in *A*. *thaliana* [56]. PRL1 is conserved throughout the lineage and the lands plants have its paralog—PRL2—as well.

The bioinformatics based characterization and functional association mapping resulted in an extensive database for microalgae and plant DWD; however, the predicted function of un-annotated DWD proteins still needs to be confirmed. Insertion mutational approaches may improve our understanding of the exact functions of DWD explored in this study.

## Supporting information

S1 Table List of other domain accompanying DWD domain in WD40 protein. (PDF)

S2 Table DWD proteins from *Arabidopsis thaliana* with description. This database was used as reference to characterize DWDs recognized in other viridiplante.
(PDF)

S3 Table Blast results of query DWD with *Arabidopsis thaliana* DWD proteins. (XLS)

S4 Table Blast % similarity of DWD proteins in microalgae and land plants with *Arabidopsis thaliana* DWD proteins. (XLS)

S1 Fig Zoom-in phylogenetic tree constructed based on D1 DWD motifs in *Arabidopsis thaliana*. (XLS)

S2 Fig Zoom-in complete phylogenetic tree generated from D1 DWD motifs of viridiplantae lineage. (XLS)

S1 File Database of identified DWD proteins in the studied viridiplante lineages. This includes the information on DWD domain sequences and position of the motif in the protein (D1, D2 and D3). Further, DWD characterization with information on other domains, if present, is provided.
(XLS)
