# Supplementary material for: Evolution of DDB1-binding WD40 (DWD) in the viridiplantae
Source: PLoS One. 2018 Jan 2;13(1):e0190282. doi: 10.1371/journal.pone.0190282 (PMC5749748; doi:10.1371/journal.pone.0190282)
Supplement: S1 Table — (PDF) [file pone.0190282.s001.pdf]

S1 Table. List of other domain accompanying DWD domain in WD40 protein.

S. No.	Other Domain	Description
1.	AAA	ATPase Associated with diverse cellular Activities
2.	ACE1-Sec16	Ancestral coatomer element 1 (ACE1) of COPII coat complex assembly protein Sec16
3.	Aldose_epim	Aldose 1-epimerase
4.	APC4	Anaphase Promoting Complex Subunit 4
5.	ARM	Armadillo repeat
6.	BAR	Bin/Amphiphysin/Rvs-homology
7.	BBP1_C	Bilin Binding Protein domain
8.	BEACH	Beige and Chediak-Higashi domain
9.	BING4CT	BING4 family of nucleolar WD40 repeat protein
10.	BOP1NT	Block Of Proliferation 1
11.	Bromo_WDR9_I_like	Bromo-domain
12.	bZIP	Basic Leucine Zipper Domain
13.	CAF1C_H4-bd	Histone-binding protein RBBP4 or subunit C of CAF1 complex
14.	CARP	Domain in CAPs (cyclase-associated proteins) and X-linked retinitis pigmentosa 2 gene product.
15.	CENP-f_leu_zip	Leucine-rich repeats of kinetochore protein Cenp-F/LEK1
16.	CEP19	Centrosomal Protein 19
17.	CTLH	C-terminal LisH motif
18.	DENN	Differentially Expressed in Neoplastic Versus Normal Cells Domain
19.	DHR2_DOCK	Dock Homology Region 2, a GEF domain, of Dedicator of Cytokinesis
20.	DEXDc	DEAD-like helicases superfamily
21.	DOR1	Dor1-like family involved in vesicle targeting
22.	DSHCT	DOB1/SK12/helY-like DEAD box helicases
23.	DUF3337	Domain of unknown function
24.	EFh	EF-hand, calcium binding motif
25.	ELAV_HUD_SF	ELAV (Embryonic lethal abnormal visual protein)/HuD (human paraneoplastic encephalomyelitis antigen D) family splicing factor
26.	FAP148	FKBP-associated protein
27.	Fcf2	Nucleolar protein domain involved in the early steps of 35S rRNA processing
28.	Fis1	Mitochondrial fission 1 protein
29.	FLiJ	Flagellar Biosynthesis Chaperone
30.	FLN	Filamin-type immunoglobulin-like domains
31.	FYDLN_acid	Protein of unknown function
32.	GEMIN8	Gem Nuclear Organelle Associated Protein 8
33.	Glyco_tranf_GTA_type	Glycosyl transferases with a common (Glycosyltransferase family A) GT-A

34.	GrpE	Nucleotide exchange factors for DnaK-type Hsp70s
35.	GYD	Uncharacterized bacterial protein
36.	HEAT	Huntington, Elongation Factor 3, PR65/A, TOR
37.	HELICc	Helicase Superfamily C-Terminal Domain
38.	Hira	Histone Cell Cycle Regulator
39.	HP_PGM_like	Histidine phosphatase domain found in phosphoglycerate mutases
40.	HslU	ATP-dependent protease ATP-binding subunit
41.	HTH_MerR-SF	Helix-Turn-Helix DNA binding domain of transcription regulators from the MerR superfamily
42.	Katanin_con80	Con80 domain of katanin-p60, the catalytic ATPase.
43.	Kelch	Kelch is a 50-residue motif, and represents one beta-sheet blade, and several of these repeats can associate to form a beta-propeller.
44.	Lebercilin	Ciliary protein causing Leber congenital amaurosis disease
45.	LisH	Lissencephaly type-1-like homology motif
46.	LRR_8	Leucine rich repeat
48.	MgtE_N	MgtE intracellular N domain involved in magnesium binding
49.	MPLKIP	M-Phase Specific PLK1 Interacting Protein
50.	MTBP_C	MDM2 (E3 ubiquitin-protein ligase that mediates ubiquitination of p53/TP53)-binding protein
51.	NLE	This domain is located N-terminal to WD40 repeats, and found in the microtubule-associated protein.
52.	NUC153	Small domain is found in a a novel nucleolar family
53.	NUP	Nucleoporin protein
54.	OmpH	Outer membrane protein
55.	OM_channels	Outer Membrane channels; Porin superfamily
56.	zfPARP	Poly (ADP-ribose) polymerase and DNA-Ligase Zn-finger region
57.	PFU	Domain appears to be unique to the PLAA family of proteins
58.	Phage_tai	Phage tail domain
59.	PKc like	Protein Kinase C domain
60.	PLAT	Polycystin-1, Lipoxygenase, Alpha- Toxin
61.	PLN	Phospholamban
62.	PUL	Alpha-helical Ub-associated domain
63.	Rab5ip	Rab5-interacting protein
64.	Raptor_N	Raptor N-terminal CASPase like domain
65.	Rav1p_c	RAVE protein 1 C terminal
66.	RCSD1	RCSD domain containing 1
67.	REC	cheY-homologous receiver domain
68.	Ribosomal_L23	Ribosomal protein L23
69.	RING	Really Interesting New Gene finger domain
70.	RRM	RNA recognition motif domain

71.	rRNA_proc-arch	rRNA-processing arch domain
72.	SANT	Nuclear receptor co-repressors and in the subunits of many chromatin-remodelling complexes
73.	SAM	Sterile alpha motif
74.	iSH2_PI3K_IA_R	Inter-Src homology 2 (iSH2) helical domain of Class IA Phosphoinositide 3-kinase Regulatory subunits
75.	SOF1	Essential for cell growth and is a component of the nucleolar rRNA processing machinery
76.	SOUL	SOUL heme-binding protein
77.	SFM	Splicing factor motif
78.	SPEC	Spectrin repeats
79.	SPS2	SPorulation Specific
80.	SRA1	Steroid receptor RNA activator
81.	STKc_Vps15	Serine/threonine-protein kinase VPS15
82.	TAF5_NTD2	The second conserved N-terminal region of TATA Binding Protein (TBP) Associated Factor 5 (TAF5), involved in forming Transcription Factor IID (TFIID)
83.	TBCC	Tubulin Binding Cofactor C
84.	TMF_TATA_bd	The C-terminal conserved coiled coil region of a family of TATA element modulatory factor 1
85.	TolA_full	Import membrane protein
86.	TPR	Tetratricopeptide repeats
87.	UBox	E3 ligases
88.	vWFA	von Willebrand factor (vWF) type A domain
