# Supplementary material for: Evolution of DDB1-binding WD40 (DWD) in the viridiplantae
Source: PLoS One. 2018 Jan 2;13(1):e0190282. doi: 10.1371/journal.pone.0190282 (PMC5749748; doi:10.1371/journal.pone.0190282)
Supplement: S2 Table — This database was used as reference to characterize DWDs recognized in other viridiplante. (PDF) [file pone.0190282.s002.pdf]

S2 Table. DWD proteins from *Arabidopsis thaliana* with description. This database was used as reference to characterize DWDs recognized in other viridiplante.

S.No.	NCBI Accession #	Characterization
1	NP_172528.2	transducin/WD40 repeat-like superfamily protein
2	NP_172582.2	WD40 domain-containing protein
3	NP_172998.1	periodic tryptophan protein 2
4	NP_173405.2	UV-B response protein-like protein CSAat1b
5	NP_173478.2	transducin/WD-40 repeat-containing protein
6	NP_174105.2	DDB1a interacting protein ATCSA-1
7	NP_174220.1	peroxin 7
8	NP_174226.1	WD40 domain-containing protein
9	NP_175413.1	cell cycle arrest protein BUB3
10	NP_175717.1	SPA1-related 4 protein
11	NP_176316.4	protein DWD hypersensitive to ABA 3
12	NP_176683.1	DDB1a-binding WD40 domain protein
13	NP_177513.2	RNA splicing protein SMU1
14	NP_177753.1	WD repeat-containing protein DWA2
15	NP_178182.1	Rae1-like protein
16	NP_178186.1	drought-sensitive 1 protein
17	NP_178242.3	nucleotide binding protein
18	NP_178635.1	WD40 domain-containing protein
19	NP_179269.1	WD-40 repeat-containing protein MSI2
20	NP_179544.1	transducin family protein / WD-40 repeat family protein
21	NP_179623.1	WD40 domain-containing protein
22	NP_179795.2	protein LETHAL WITH SEC THIRTEEN 8-2
23	NP_180854.1	E3 ubiquitin-protein ligase COP1
24	NP_181681.1	splicing factor PRP4-like protein LACHESIS
25	NP_181905.1	transducin/WD40 repeat-like superfamily protein
26	NP_182151.1	TGF-beta receptor interacting protein 1
27	NP_182157.2	protein SUPPRESSOR OF PHYA-105
28	NP_182179.3	transducin family protein / WD-40 repeat family protein
29	NP_182320.1	transducin family nucleolar protein with WD40 repeats
30	NP_187664.2	transducin/WD40 domain-containing protein
31	NP_188434.2	WD40 domain-containing protein
32	NP_188442.1	protein LETHAL WITH SEC THIRTEEN 8-1
33	NP_188710.1	polycomb group protein FERTILIZATION-INDEPENDENT ENDOSPERM
34	NP_190148.1	DWD motif protein
35	NP_190487.1	root initiation defective 3 protein

36	NP_190761.1	transducin/WD40 domain-containing protein
37	NP_192095.2	WD40 domain-containing protein
38	NP_192182.1	transducin/WD40 domain-containing protein
39	NP_192211.2	WD40 domain-containing protein
40	NP_192450.1	WD40-repeats containing protein YAOZHE
41	NP_192849.4	protein SPA1-related 2
42	NP_193325.1	protein pleiotropic regulatory locus 1
43	NP_193845.2	U3-55K-like protein 2
44	NP_193883.2	WD40 repeat family protein
45	NP_194667.1	phosphoinositide-3-kinase, regulatory subunit 4, p150
46	NP_194702.2	nucleosome/chromatin assembly factor group C5
47	NP_194712.1	SKI8 like WD40 repeat-containing protein
48	NP_194715.2	WD repeat protein DSE1
49	NP_195154.2	WD40 domain-containing protein
50	NP_195172.1	guanine nucleotide-binding protein subunit beta
51	NP_195231.1	WD-40 repeat-containing protein MSI3
52	NP_195240.2	WD repeat-containing protein 42A
53	NP_195797.3	regulatory-associated protein of TOR 2
54	NP_196796.5	WD40 domain-containing protein
55	NP_196852.3	Flowering time control protein FY
56	NP_196909.1	transducin/WD40 repeat-like superfamily protein
57	NP_196957.1	transducin/WD40 domain-containing protein
58	NP_197059.1	WD-40 repeat family protein
59	NP_197239.2	transducin/WD40 domain-containing protein
60	NP_197492.1	WD40 domain-containing protein
61	NP_197734.2	Katanin p80 WD40 repeat-containing subunit B1-1
62	NP_197859.4	WD40 domain-containing protein
63	NP_198109.1	cell division cycle 20.5, cofactor of APC complex
64	NP_200038.1	UV-B induced transducin protein
65	NP_200094.1	Notchless protein homolog
66	NP_200263.2	WD40 domain-containing protein
67	NP_200424.1	THO complex subunit 3
68	NP_200631.1	histone-binding protein MSI1
69	NP_200684.2	protein DAMAGED DNA-BINDING 2
70	NP_201106.1	WD40 domain-containing protein
71	NP_201533.1	histone deacetylation-related WD-40 repeat protein
72	NP_564469.1	transducin/WD-40 repeat-containing protein
73	NP_564699.1	transducin/WD-40 repeat-containing protein
74	NP_564965.1	transducin/WD-40 repeat-containing protein
75	NP_565168.4	transducin/WD40 repeat-like superfamily protein
76	NP_565456.2	WD-40 repeat-containing protein MSI4

77	NP_565594.3	transducin family protein / WD-40 repeat family protein
78	NP_566111.1	transducin/WD-40 repeat-containing protein
79	NP_566246.1	protein lateral root stimulator 1
80	NP_566335.1	Regulatory-associated protein of TOR 1
81	NP_566453.1	transducin/WD40 domain-containing protein
82	NP_566557.1	protein pleiotropic regulator PRL2
83	NP_566620.1	transducin family protein / WD-40 repeat family protein
84	NP_566644.1	cell cycle arrest protein BUB3
85	NP_566822.1	transducin/WD40 repeat-like superfamily protein
86	NP_567810.5	WD repeat and SOF domain-containing protein 1
87	NP_568042.1	transducin/WD40 domain-containing protein
88	NP_568194.2	transducin/WD40 repeat-like superfamily protein
89	NP_568242.1	WD and tetratricopeptide repeats protein 1
90	NP_568338.2	WD-40 repeat protein TOZ
91	NP_568435.2	WD repeat-containing protein RUP2
92	NP_568838.1	transducin/WD40 domain-containing protein
93	NP_568993.1	transducin/WD40 domain-containing protein
94	NP_569031.1	transducin/WD40 domain-containing protein
95	NP_683567.1	protein SPA1-related 3
96	NP_849494.2	guanine nucleotide-binding protein subunit beta
97	NP_849536.1	WD40 domain-containing protein
98	NP_849802.1	SPA1-related 4 protein
99	NP_849989.1	WD repeat-containing protein DWA1
100	NP_850206.4	U-box protein MAC3B
101	NP_850450.1	TGF-beta receptor interacting protein 1
102	NP_850474.3	WD40 domain-containing protein
103	NP_850516.1	protein lateral root stimulator 1
104	NP_850612.1	transducin family protein / WD-40 repeat family protein
105	NP_850959.1	protein STOMATAL CYTOKINESIS-DEFECTIVE 1
106	NP_851064.1	Katanin p80 WD40 repeat-containing subunit B1-1
107	NP_851199.1	transducin/WD40 domain-containing protein
108	NP_851281.1	transducin/WD40 domain-containing protein
109	NP_973697.1	TGF-beta receptor interacting protein 1
110	NP_973836.1	periodic tryptophan protein 2
111	NP_974113.1	transducin/WD-40 repeat-containing protein
112	NP_974683.1	guanine nucleotide-binding protein subunit beta
113	NP_974784.1	WD-40 repeat family protein
114	NP_974804.4	protein serine/threonine kinase
